# Timing of the repeat thyroid fine-needle aspiration biopsy: does early repeat biopsy change the rate of nondiagnostic or atypia of undetermined significance cytology result?

**DOI:** 10.1007/s12020-024-03953-7

**Published:** 2024-07-10

**Authors:** Fatma Dilek Dellal Kahramanca, Muhammet Sacikara, Aydan Kilicarslan, Berna Ogmen, Cevdet Aydin, Oya Topaloglu, Reyhan Ersoy, Bekir Cakir

**Affiliations:** 1 Department of Endocrinology and Metabolism, Ankara Bilkent City Hospital, University of Health Sciences, Ankara, Turkey; 2 Department of Endocrinology and Metabolism, Ankara Bilkent City Hospital, Ankara, Turkey; 3 Department of Pathology, Ankara Yildirim Beyazit University, Ankara, Turkey; 4 Department of Endocrinology and Metabolism, Ankara Yildirim Beyazit University, Ankara, Turkey

**Keywords:** AUS, Early thyroid rebiopsy, Nondiagnostic, Thyroid nodule cytology

## Abstract

**Purpose:**

To determine whether early repeat fine needle aspiration biopsy (FNA) has an effect on adequate or atypia of undetermined significance (AUS) cytology rates in thyroid nodules with inadequate or AUS result in the first FNA.

**Methods:**

Nodules of patients who underwent repeat biopsy due to insufficient or AUS cytology between 2019–2022 were included. Data of the patients and ultrasonographic, cytological and histopathological results of the nodules were recorded. Additionally, the time between the two biopsies was noted. The first was called “initial” and the second was called “rebiopsy”. Five different paired groups were formed according to the time between two consecutive biopsies; before and after 1 month, 45 days, 2 months, 3 months, and 6 months. The groups were compared in terms of adequate and AUS cytological results.

**Results:**

We evaluated 1129 patients with 2187 nodules undergoing FNAB. After excluding nodules with one FNA result and/or missing data, 966 nodules of 628 patients who underwent FNA at least twice were included. Initial cytology was nondiagnostic (ND) in 665 (30.4%) and AUS in 301 (13.8%) nodules. The mean age of the patients was 52.0 ± 11.9 years, and the female sex ratio was 78.8% (n = 495). There were no differences in adequate or AUS rebiopsy results according to the different time interval groups (p > 0.05 for all). AUS result was statistically insignificantly more frequent in nodules with initially AUS nodules when rebiopsy was performed before 1 month in comparison to after 1 month (53.8%, 27.1%; p = 0.054). Accuracy of rebiopsy was also similar in the time intervals groups (p > 0.05 for all).

**Conclusion:**

In patients with inadequate or AUS initial biopsy, the rate of adequate or AUS cytology results at rebiopsy did not vary with the timing of repeat biopsy indicating that there may be no need to wait 1 month for a repeat biopsy. In patients with suspicious nodules, biopsy might be repeated before 1 month.

## Introduction

Thyroid fine-needle aspiration biopsy (FNA) is a widely used, safe and an inexpensive method for detecting thyroid cancer in patients with thyroid nodule [1]. However, FNA is reported as nondiagnostic (ND)/suboptimal or indeterminate/atypical in 35–45% of the materials [2]. Biopsy repetition was recommended because these nodules have a mean malignancy potential up to 2 and 16%, respectively [3]. Additionally, repeat aspiration of ND nodules will result in diagnostic results in 60–80% of cases, especially in the nodules with a smaller cystic component [4]. If repeat FNA results are benign, it might lessen the number of futile thyroidectomies. Although molecular testing has been suggested for nodules with atypia of undetermined significance (AUS) cytology, it has still limited availability because of higher costs and requirement for experienced staff. Also there are clinical concerns regarding its lower positive predictive value (PPV) and specificity (45–58, and 12–78%, respectively) although its sensitivity and negative predictive value (NPV) is higher (84–98, 91–93%, respectively) [5]. Therefore, repeat FNA is a more viable and common approach used to achieve definitive diagnosis in most countries.

Repetition of FNA was advised to perform no earlier than 3 months in order to avoid the possibility of reactive/reparative cellular changes being interpreted as false positive in the 2009 The Bethesda System for Reporting Thyroid Cytopathology (TBSRTC) [6]. The National Cancer Institute also made similar suggestions [7]. The American Association of Clinical Endocrinologists (AACE) recommends a waiting period of at least 1 month before reaspiration [8]. In studies investigating histological changes in needle trace [9–13], reactive changes (large nuclei, nuclear groove and nuclear clarification) were detected which peaked 20–40 days after biopsy were detected [10]. The recommendation for a 3-month waiting period is largely based on the results of these histopathological studies and this peak period. However, there is no data indicating that repeating the biopsy within 3 months increases the false positivity rate.

There is a suspicion about the necessity of the 3-month waiting period in the last American Thyroid Association (ATA) guideline [14]. Also, it was stated that if clinical and ultrasonographic features are suspicious for malignancy, a shorter waiting time may be more appropriate [8, 14]. However, no specific time recommendation has been provided. Additionally, the recent TBSRTC denoted that data are slightly conflicting about the interval for a repeat FNA and delaying a repeat FNA for 3 months seems to be less essential. It also avoided giving any specific period regarding the timing of rebiopsy [3]. Both the ATA guideline and the recent TBSRTC made these suggestions based on a small number of studies, which were performed with a small number of cases [15–17]. Controversial results exist about the effect of repeat FNA interval on cytological results in the literature [1, 18–23].

In this study, we aimed to determine whether early repetition of FNA has an effect on adequate (diagnostic) or AUS cytology rate in thyroid nodules with initially ND or AUS cytology results.

## Material and methods

Thyroid FNA records at our outpatient clinic were screened from 2019 to 2022 under the institutional ethics committee approval. Nodules that were rebiopsied due to ND or AUS cytology were included in this retrospective study. The study was conducted in agreement with the revised Declaration of Helsinki [24].

Demographic (age, gender) and clinical (diagnosis of patients, thyroid functional status, use of thyroid-related medications, histories of thyroidectomy and radioactive iodine treatment and radiotherapy to the neck) data of the patients were recorded.

Blood samples for thyroid hormones and also thyroid autoantibodies (anti-thyroid peroxidase and anti-thyroglobuline) were collected after an 8 h fasting in the morning within the last month before the FNA. They were studied by Siemens Atellica® Solution IM 1600 (Siemens Healthineers) biochemistry analyzer with their original kits. Normal reference ranges were 0.55–4.78 mU/L for thyroid stimulating hormone (TSH), 2.3–4.2 ng/L for free triiodothyronine, 0.89–1.76 ng/dL for free thyroxine, <60 U/mL for anti-thyroid peroxidase antibody, and <1.3 IU/mL for anti-thyroglobuline antibody. Patient was classified as subclinical hypothyroidism, subclinical hyperthyroidism, and euthyroid in state of normal level of free thyroid hormones when TSH was between above the upper limit of the reference level and 10 U/L, below the reference value, and in normal reference range, respectively. Calcitonin analysis was performed with a Siemens IMMULITE® 2000 automated chemiluminescence immunoassay analyzer using its standard assay kit. Its sensitivity was 2 pg/mL and reference values were up to 5 pg/mL for women and up to 8.4 pg/mL for men.

Thyroid ultrasonography (US) was carried on all patients by experienced endocrinologist or endocrinology residents collaboration with experienced endocrinologist using a high frequency (10–15 MHz) linear array probe (Hitachi Hivision Avius with EUP‐L74M, Hitachi Aloka Medical, Tokyo, Japan). US features of nodules (diameter, texture, echogenicity, border regularity, presence of micro- and/or macrocalcification, presence of halo) were recorded. When nodule diameter ≤10 mm and >10 mm, the nodule was defined as micronodule and macronodule respectively. Suspicious US features were considered microcalcifications, hypoechoic appearance, irregular borders, taller-than-wide shape, and solid texture. Thyroid Imaging Reporting and Data System (TI-RADS) categories were determined according to suspicious US features as previously suggested by Kwak et al. [25].

FNA was performed under US guidance by experienced endocrinologist or endocrinology residents with assistance of experienced endocrinologist in agreement with criteria of the last ATA guideline [14]. Informed consent was obtained from each patient before the procedure. Logic Pro 200 GE and 7.5 MHz probes (Kyunggigo, Korea) were used for FNA. Local anesthesia was not applied routinely. Nonaspirating FNA technique was done with a 23–25-gauge needle. The cytopathologist did not accompany the procedure in the biopsy room due to lack of staff. Cytological materials were air-dried, stained with May-Grünwald-Giemsa, and classified according to the Bethesda system as benign, ND, AUS, follicular neoplasm (FN), suspicious for malignancy and malignant [3]. The number of nodules biopsied in each patient was recorded. The time interval between the two biopsies was noted. The first biopsy was called “initial”, and the second was called “rebiopsy” or “repeat FNA”. Five different paired groups were formed according to the interval between two consecutive biopsies: (1) ≤/> 1 month, (2) ≤/> 45 days, (3) ≤/> 2 months, (4) ≤/> 3 months, and (5) ≤/> 6 months.

Rebiopsy materials were evaluated according to two different paired cytological groups. Nodules with ND cytology and those with other than ND cytology were categorized as “ND/inadequate” and “diagnostic/adequate”, subsequently. Secondly, nodules were grouped as “AUS” and “non-AUS” when their biopsy material resulted in AUS and all cytology results other than AUS, respectively. The time groups were compared in terms of the results of these two groups.

Histopathological assessment was made in accordance with the 2017 World Health Organization criteria [26]. Benign and malignant results were recorded for all patients who underwent thyroidectomy. Malignant histopathological outcomes derived from parenchyma or other than biopsied nodules were not registered as malignant results. The false negative result was defined if benign cytology was presented with the malign histopathology whereas the false positive result was vice versa (malignant cytology resulted in benign histopathology).

SPSS package (SPSS, Chicago, Ill., USA, version 20) was used for statistical analysis. The Shapiro-Wilk test was examined for investigating the normality of the distributions of the continuous variables. Patient age was expressed as mean ± standard deviation. Quantitative variables were assessed with median (IQR [interquartile range], 25–75th percentiles). Categorical variables were analyzed by chi square test or Fisher’s exact test, as required, and expressed as the number and percent (%). Mann-Whitney U test was used for the continuous variables. In the diagnosis of malignant tumors, the sensitivity, specificity, PPV and NPV, and accuracy of rebiopsy results were calculated in comparison to histopathological results. p < 0.05 was considered statistically different.

## Results

We screened 1129 patients with 2187 nodules undergoing FNA. Single FNA was performed on 1080 (49.4%) nodules, while 2 or more FNAs were performed on 1107 (50.6%) nodules. After excluding nodules with one FNA result and/or missing data, the study finally consisted of 966 nodules of 628 patients. Initial cytology was ND in 665 (30.4%) and AUS in 301 (13.8%) nodules. The mean age of the patients was 52.0 ± 11.9 years. There was female dominance (78.8%, n = 495). Thyroidectomy and radioiodine treatment history existed in 34 (5.4%) and 2 (0.3%) patients, respectively. None of the patients had radiotherapy history to the neck region. While 84 (13.4%) of the patients had subclinical hypothyroid and 131 (9.2%) were subclinical hyperthyroid, the remaining patients were euthyroid. Seventy nine patients (12.6%) received levothyroxine and 51 (8.1%) patients received anti-thyroid drug treatment. Positivity of anti-thyroid peroxidase and anti-thyroglobuline antibodies were present in 130 (21.0%) and 142 (23.2%) patients, respectively. Calcitonin was detectable in 4.8% (n = 29). Median calcitonin level was 7.7 (6.1–12.4) pg/mL in women and 10.8 (8.6–13.9) pg/mL in men with calcitonin positivity.

On the follow-up, three or more FNA were carried out in 395 nodules because of an increase in nodule dimension or to ensure cytology in patients unwilling or unable to perform the thyroidectomy. One hundred twenty patients with a total of 249 nodules were operated. Thirty two patients (26.7%) with 49 (19.7%) nodules had malignant histopathology.

US features of nodules according to adequacy and AUS result in second biopsy were given in Table 1. Eighty eight (9.1%) nodules were micronodules while 878 (90.9%) were macronodules. Diagnostic nodules had more frequently solid structure, isoechoic appearance and halo presence than ND ones (94.3, 88.4%, p = 0.001; 80.2, 65.5%, p < 0.001; 18.9, 12.6%, p = 0.011; respectively). The ND group more prominently consisted of heterogene and TI-RADS 3 nodules than the diagnostic group (25.2, 13.8%, *p* < 0.001; 8.9, 4.5%, p = 0.007; respectively). AUS nodules were less frequently heterogeneous and more frequently included halo than the non-AUS nodules (12.6, 19.5%, *p* = 0.027; 26.6, 13.8%, *p* < 0.001). Other US features were comparable between groups.

**Table 1:** Ultrasonography features of the nodules according to adequacy and atypia of undetermined significance result in repeat biopsy

Ultrasonography features	Adequacy			AUS		
	Nondiagnostic/Inadequate (n = 372, 38.5%)	Diagnostic/Adequate (n = 594, 61.5%)	*p*	AUS (n = 203, 21.0%)	Non-AUS (n = 763, 79.0%)	*p*
Dimensions (mm)						
Anteroposterior	10.9 ± 6.6	10.7 ± 6.0	0.709	11.0 ± 6.4	10.7 ± 6.2	0.569
Transverse	14.7 ± 9.4	14.6 ± 8.5	0.841	15.3 ± 9.0	14.5 ± 8.8	0.252
Longitudinal	17.4 ± 12.2	17.2 ± 10.4	0.811	17.9 ± 11.3	17.1 ± 11.1	0.376
Micronodule, n (%)	34 (9.1)	54 (9.1)	0.986	18 (8.9)	70 (9.2)	0.888
Solid structure, n (%)	329 (88.4)	**560 (94.3)**	**0.001**	190 (93.6)	699 (91.6)	0.354
Echogenicity (n = 893), n (%)			**<0.001**			0.086
Hypoechoic	31 (9.3)	34 (6.1)	0.072	15 (7.9)	50 (7.1)	0.851
Isoechoic	218 (65.5)	**449 (80.2)**	**<0.001**	152 (79.6)	515 (73.4)	0.080
Heterogene	**84 (25.2)**	77 (13.8)	**<0.001**	24 (12.6)	**137 (19.5)**	**0.027**
Irregular border (n = 448), n (%)	144 (84.7)	246 (88.5)	0.247	70 (81.4)	320 (88.4)	0.082
Calcification, present, n (%)	55 (14.8)	83 (14.0)	0.726	27 (13.3)	111 (14.5)	0.652
Microcalcification, n (%)	16 (4.3)	35 (5.9)	0.282	10 (4.9)	41 (5.4)	0.800
Macrocalcification, n (%)	43 (11.6)	57 (9.6)	0.330	21 (10.3)	79 (10.4)	0.997
Taller than wider, n (%)	28 (7.5)	58 (9.8)	0.232	18 (8.9)	68 (8.9)	0.980
Halo presence, n (%)	47 (12.6)	**112 (18.9)**	**0.011**	**54 (26.6)**	105 (13.8)	**<0.001**
TI-RADS categories			**0.043**			0.280
3	**33 (8.9)**	27 (4.5)	**0.007**	11 (5.4)	49 (6.4)	0.717
4a	164 (44.1)	257 (43.3)	0.802	100 (49.3)	321 (42.1)	0.066
4b	142 (38.2)	250 (42.1)	0.228	72 (35.5)	320 (41.9)	0.095
4c	32 (8.6)	60 (10.1)	0.528	20 (9.9)	72 (9.4)	0.903
5	1 (0.3)	0 (0.0)		0 (0.0)	1 (0.1)	

*AUS* atypia of undetermined significance, *TI-RADS* Thyroid Imaging Reporting and Data System. Statistically significant results were shown in bold font

Rebiopsy and histopathology results according to initial FNA results are presented in Table 2. Rebiopsy results were ND in 372 (38.5%) nodules and AUS in 203 (21.0%) nodules. Repeat FNAs resulted in benign in 264 (39.7%) of initial ND nodules and 116 (38.5%) of initial AUS nodules. Initially ND nodules yielded ND in 281 (42.3%) and AUS in 118 (17.7%) in rebiopsy. For initial AUS nodules, rates were 91 (30.2%) for ND and 85 (28.2%) for AUS in repeat FNA. ND results in repeat FNA were inadequate for the presence of blood material in 145 (39.0%) nodules, virtually acellular material in 192 (51.6%) nodules, cyst fluid in 52 (14.0%) nodules, and clotting artifact in 72 (19.4%) nodules. In the cytology report, there was no detailed explanation of the reason for the ND result in 122 (32.8%) nodules. Repeat FNA were performed in a median of 62 (47–91) days. It was done ≤1 month in 50 (5.2%) nodules, ≤45 days in 225 (23.3%) nodules, ≤2 months in 461 (47.7%) nodules, ≤3 months in 722 (74.7%) nodules and ≤6 months in 888 (91.9%) nodules.

**Table 2:** Repeat fine needle aspiration results according to initial fine needle aspiration cytology and histopathology results

	Initial FNA			Histopathology*	
	Nondiagnostic/Inadequate (n = 665)	AUS (n = 301)	Total (n = 966)	Malignant (49/249)	Benign (200/249)
Repeat FNA	n (%)	n (%)	n (%)	n (%)	n (%)
Benign	264 (39.7)	116 (38.6)	380 (39.3)	2 (2.8)	69 (97.2)
Nondiagnostic/Inadequate	281 (42.3)	91 (30.2)	372 (38.5)	15 (14.9)	86 (85.1)
AUS	118 (17.7)	85 (28.2)	203 (21.0)	21 (31.8)	45 (68.2)
FN	0 (0.0)	2 (0.7)	2 (0.3)	2 (100.0)	0 (0.0)
Suspicious for malignancy	0 (0.0)	1 (0.3)	1 (0.1)	1 (100.0)	0 (0.0)
Malignant	2 (0.3)	6 (2.0)	8 (0.8)	8 (100.0)	0 (0.0)

*FNA* fine-needle aspiration biopsy, *AUS* atypia of undetermined significance, *FN* follicular neoplasm

*% according to repeat FNA results

**Fig. 1:**
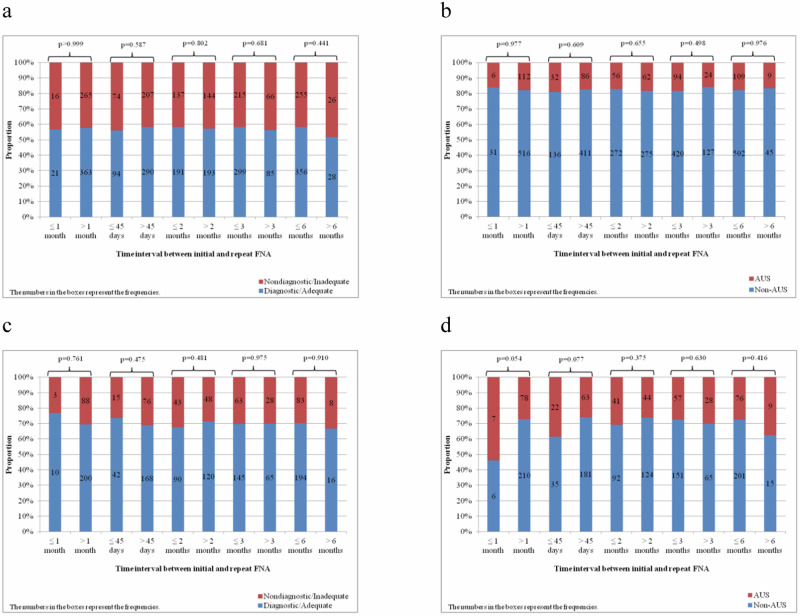
Repeat biopsy results according to the time interval. **a** Adequacy in patients with initially nondiagnostic nodules. **b** Atypia of undetermined significance results in patients with initially nondiagnostic nodules. **c** Adequacy in patients with initially atypia of undetermined significance biopsy results. **d** Atypia of undetermined significance results in patients with initially atypia of undetermined significance biopsy results

Cytological results in second biopsy were not different according to any time interval between initial and second biopsy in patients with initially either ND or AUS FNA as shown in Fig. 1. Repeat AUS results were more frequent before 1 month compared to after 1 month but the difference was not statistically significant (53.8 vs 27.1%, p = 0.054).

Evaluating the histological results of 49 malignant nodules, 38 nodules (77.6%) were papillary thyroid carcinoma (PTC), 6 (12.2%) were non-invasive follicular thyroid neoplasm with papillary-like nuclear features (NIFT-P), 3 (6.1%) were well-differentiated tumor of uncertain malignant potential and 2 (4.1%) were medullary thyroid carcinoma. Overall malignancy rate was 12.1% (19/157) and 32.6% (30/92) for initial ND and AUS nodules, respectively. There were no false positive results but two false negative results (2/49, 4.1%) were present. Initial FNAs of both false negative nodules were AUS and their rebiopsy performed after 45 days (days 55 and 60) had resulted in benign cytology. TI-RADS categories of them were 4a and 4b. Operation indications were giant nodules. Histopathologic results were PTC and NIFT-P.

The sensitivity, specificity, PPV, NPV, and accuracy of rebiopsy results were given in Table 3. Rebiopsy at all time intervals were 100% predictive for malignant histopathology. Whereas predictive values for benign histopathology were 100, 100, 96.1, 96.9, and 97.2% for ≤1 month, ≤45 days, ≤2 months, ≤3 months and ≤6 months, respectively.

**Table 3:** Diagnostic performance of rebiopsy according to time intervals

Time interval	*TP*	*TN*	*FP*	*FN*	*Sensitivity (%)*	*Specificity (%)*	*PPV (%)*	*NPV (%)*	*Accuracy (%)*	p*
≤1 month	2	3	0	0	100.0	100.0	100.0	100.0	100.0	1.000
>1 month	7	66	0	2	77.8	100.0	100.0	97.1	97.3	
≤45 days	5	22	0	0	100.0	100.0	100.0	100.0	100.0	0.547
>45 days	4	47	0	2	66.7	100.0	100.0	95.9	96.2	
≤2 months	8	49	0	2	80.0	100.0	100.0	96.1	96.6	1.000
>2 months	1	20	0	0	100.0	100.0	100.0	100.0	100.0	
≤3 months	9	63	0	2	81.8	100.0	100.0	96.9	97.3	1.000
>3 months	0	6	0	0	–	100.0	–	100.0	100.0	
≤6 months	9	69	0	2	81.8	100.0	100.0	97.2	97.5	–
>6 months	0	0	0	0	–	–	–	–	–	

*TP* true positive, *TN* true negative, *FP* false positive, *FN* false negative, *PPV* positive predictive value, *NPV* negative predictive value

*p calculated for accuracy

## Discussion

We found that the rate of adequate or AUS cytology results at rebiopsy were not different in various time intervals for initially ND or AUS nodules. AUS result in rebiopsy was statistically insignificantly more frequent in nodules with initially AUS nodules when FNA was performed before 1 month.

Conception of avoiding rebiopsy no earlier than 3 months is mainly grounded in the results of histopathological studies. Cytological studies were inconclusive and mostly did not support this conception although they were conducted with a small number of patients [1, 18–23]. So, there is a hesitation about waiting for 3 months for rebiopsy according to the world-wide accepted guidelines and results of some literature [1, 3, 8, 14–17, 19, 21, 22].

Our study showed that FNA of initial AUS nodules had similar rebiopsy results in all time intervals. AUS nodules yielded the same cytology more frequently when rebiopsy was performed before 1 month compared to after 1 month but the difference was statistically insignificant. Diagnostic results for rebiopsy were similar in all time intervals for initial AUS nodules. Most of the studies showed that repeat sampling at 3 and/or 6 month intervals after an initial AUS did not affect the rate of AUS results and diagnostic yield [15, 18, 19]. Silva et al. found a similar rate of AUS result in repeat FNA within 3 months in a study involving 246 nodules with ND and AUS results. Similar to our result, but in the 3 months of the rebiopsy interval, they showed more frequent AUS results without statistical significance although their study was performed with a limited number of nodules that are AUS on rebiopsy. Overall cytological results (diagnostic yield) and the rate of determinant cytology were not different from before and after both 3 and 6 months [18]. In another study including 51 nodules, Nagarkatti et al. also showed similar AUS rates and diagnostic yield before or after 3 months interval in initially AUS nodules [19]. Singh et al. studied with 307 nodules and divided them into four groups as ND, suboptimal, atypical, and adequate without using the Bethesda system. No significant difference was found in the distribution of results according to the timing of the 3-month rebiopsy interval. Diagnostic yield and accuracy were similar in rebiopsy [15]. In addition to these studies, Soutiro et al. found AUS nodules had comparable ND results in rebiopsy before or after 3 or 6 months as in our study [23]. Only Valerio et al. reported dissimilar results for the 3 month interval with 143 initially AUS nodules. They emphasized that higher diagnostic resolution, which was defined as other than ND or AUS cytology, can be obtained 2.5 times more likely if rebiopsy is performed after 3 months [20].

In the present study, the time interval between FNAs for previously ND nodules did not alter the rate of ND or AUS results. Most of the studies in the literature supported our result [1, 15–17, 20–22]. On the other hand, Souteiro et al. found that FNA performing before 3 months for initially ND nodules (n = 233) had nearly 5 times more chance of a second ND result compared to after 3 months (OR:4.74). They explained this result that previous recent FNA could provide nodules more hemorrhagic [23]. Excessive blood is one of the main contributors to ND results [27]. Our center is a high volume tertiary level hospital. We perform a considerable amount of FNAs with experienced staff with rare complications. Bloody material existed in rebiopsy of 145 ND nodules (39.0% for ND nodules, and 15.0% for all nodules) in our study. This rate was lower than the literature, in which the rate of bloody smear reached up to 60.9% of ND nodules [28]. Nonaspirating FNA technique may also contribute to the lower prevalence of abundant blood in the background [29].

US features of diagnostic thyroid nodules had lower rates of cystic proportion, macrocalcification, macronodule, and isoechogenicity [27, 30]. In our study, very similar to the literature, we detected more frequent solid structure, isoechoic appearance as well as halo in diagnostic nodules compared to ND ones.

In a study evaluating FNA-induced histological alterations, it was reported that reactive follicular cells with nuclear grooving and clearing are possible risks of repeat FNA and these alterations are reached to top within 20–40 days following FNA [10]. Although our results show that AUS cytology was more frequent within 1 month compared to after 1 month, this was not statistically significant. Likewise, other cytological research examining the effect of earlier biopsy intervals did not support this histological result that the reparative changes may affect frequency of AUS or ND results in early rebiopsy. Lee et al. showed that the 5-week interval, in addition to 10, 15, and 20-week intervals, did not alter AUS frequency on repeat FNA for initially ND nodules [16]. In another study, when ND nodules evaluated with repeat FNA within 3 months were divided into two subgroups as 45 days before and after, accuracy was similar [1].

Our series had no false positive result but two false negative results. Both nodules had initial AUS results, and their TI-RADS categories were 4a and 4b. Rebiopsy of them was performed after 45 days. Operation indication was giant nodule and histopathological diagnosis were PTC and NIFT-P. This implies that FNA should be performed without waiting in nodules having suspicious ultrasonographic features.

Our study has some limitations. Firstly, it was a retrospective study. FNAs were performed by different operators and reviewed by different cytopathologists, so interobserver variability may introduce bias. The high percentage of initial ND reports (30.4%) also might have bias. It might be related with performing FNA on nodules with cystic components even if smaller than 2 cm. Also, dissimilarities between interpretation of the materials by different pathologists may contribute to this high ratio of ND results. Several studies have reported distinct ratios of insufficient biopsies, varying from 5 to 43.1% of materials [1, 18]. For some of AUS or ND nodules, we might have preferred to perform earlier FNA due to suspicious sonographic and/or clinical factors. This might also have caused potential bias, indicating that the statistical analysis should be interpreted with caution. But this scenario is common in clinical settings and this study reflects real-life management in this aspect. Lastly, not all patients were operated. This might have contributed to the underrated false positive and/or negative ratios. The strength of our study is that, as we know, the number of nodules for each cytology group and time interval groups were the largest reported in the literature.

In conclusion; earlier rebiopsy can be performed for ND or AUS nodules depending on the choice of patient and clinician without endangering diagnostic yield and adequacy of the sample. Waiting for repeat FNA of indeterminate nodules has no diagnostic benefit but might increase patient anxiety and impede the surgical treatment of patients with thyroid cancer. FNA might be repeated without waiting, especially for nodules with suspicious US features.
