# Correction: Timing of the repeat thyroid fine-needle aspiration biopsy: does early repeat biopsy change the rate of nondiagnostic or atypia of undetermined significance cytology result?

**DOI:** 10.1007/s12020-024-03999-7

**Published:** 2024-08-23

**Authors:** Fatma Dilek Dellal Kahramanca, Muhammet Sacikara, Aydan Kilicarslan, Berna Ogmen, Cevdet Aydin, Oya Topaloglu, Reyhan Ersoy, Bekir Cakir

**Affiliations:** 1 Department of Endocrinology and Metabolism, Ankara Bilkent City Hospital, University of Health Sciences, Ankara, Turkey; 2 Department of Endocrinology and Metabolism, Ankara Bilkent City Hospital, Ankara, Turkey; 3 Department of Pathology, Ankara Yildirim Beyazit University, Ankara, Turkey; 4 Department of Endocrinology and Metabolism, Ankara Yildirim Beyazit University, Ankara, Turkey

Correction to: Endocrine (2024) 10.1007/s12020-024-03953-7

In this article the open access copyright holder name was incorrectly written as “© The Author(s), under exclusive licence to Springer Science+Business Media, LLC, part of Springer Nature 2024” but should have been “© The Author(s) 2024”.

The original article has been corrected.

